# Correction: Heritage, geographical scale and didactic potentiality: Students and teachers’ perspectives

**DOI:** 10.1371/journal.pone.0315956

**Published:** 2024-12-12

**Authors:** Ana Isabel Ponce Gea, Carlos Martínez Hernández, María Luisa Rico Gómez

The funding statement for this paper is incorrect. The correct Funding statement is as follows: Project PGC2018-094491-BC33 (Ministry of Science, Innovation and Universities, Government of Spain).
