# Supplementary material for: Heritage, geographical scale and didactic potentiality: Students and teachers’ perspectives
Source: PLoS One. 2021 May 10;16(5):e0251398. doi: 10.1371/journal.pone.0251398 (PMC8109804; doi:10.1371/journal.pone.0251398)
Supplement: S1 Appendix — (PDF) [file pone.0251398.s001.pdf]

Test on Didactic Potentiality of Heritage according to Scale (TDPHS)

From the University of Murcia, members of the “Social Science Didactics” (DICSO) Research Group are carrying out a research to contrast the perspectives of students and teachers regarding the geographical scale, heritage and their didactic potential, and to determine the relationships between geographic scales, heritage perspective and the didactic potential granted to heritage.

For this purpose, we invite you to complete this anonymous questionnaire, following the instructions for each block and each question. There are no right or wrong answers, so please feel free to express yourself.

You have 50 minutes to answer!

BLOCK 1. Personal information

Public, private or subsidized centre?	
Year	
Place of origin (municipality, district)	
Age	
Gender	

BLOCK 2. Scalar perspective

QUESTION 1

Bearing in mind the following geographical scales

- Local scale (your district and municipality; e.g., Balsapintada and Fuente Álamo)
- Regional scale (your Autonomous Community; e.g., Autonomous Community of the Region of Murcia)
- National scale (your country; e.g., Spain)
- Global scale (the world);

indicate for each one and for each statement your degree of agreement according to this scale:

(1) Strongly disagree

(2) Somewhat agree

(3) Quite agree

(4) Totally agree.

	Local scale	Regional scale	National scale	Global scale
1. I am interested.				
2. I identify myself with it.				
3. I am concerned about its problems.				
4. I would defend it.				
5. I know about it.				

BLOCK 3. Heritage perspective

QUESTION 2

Indicate which of these three options coincides, in your opinion, with the concept of heritage. Choose only one of them.

- ☐ Old, beautiful, strange.
- ☐ Our, own.
- ☐ Useful, socio-economically profitable.

QUESTION 3

For each one of the following heritage elements, categorized by typology (Tables 1, 2 and 3), indicate the adjectives with which you would describe them. Use an “x” to select the adjectives. You can choose as few or as many options as you want.

Table 1. Monuments	Local scale	Regional scale	National scale	Global scale
	**Hermitage of San Roque**	**Cathedral of Murcia**	**Cathedral of Santiago**	**Cathedral of Notre Dame**
Old				
Beautiful				
Our				
Own				
Useful				
Socio-economically profitable				

Table 2. Landscapes	Local scale	Regional scale	National scale	Global scale
	**Ravine**	**Sierra Espuña Mountains**	**Ciudad Encantada**	**Sahara Desert**
Old				
Beautiful				
Our				
Own				
Useful				
Socio-economically profitable				

Table 3. Festivities	Local scale	Regional scale	National scale	Global scale
	**San Agustín**	**Bando de la Huerta**	**Virgin of Pilar**	**Christmas**
Old				
Beautiful				
Our				
Own				
Useful				
Socio-economically profitable				

BLOCK 4. Didactic potentiality

QUESTION 4

Which of the following heritage elements would you like to see discussed in class?

Indicate, for each one, your degree of interest, according to the following scale:

(1) Not of interest

(2) Somewhat interesting

(3) Quite interesting

(4) Very interesting

**Heritage element**	**Degree of interest (from 1 to 4)**
Church of San Agustín	
Monte de la Cruz, in El Estrecho	
Roman Coliseum	
Grand Canyon of Colorado	
Trovo (spontaneous poetry of the Region of Murcia)	
Olive tree field in Córdoba	
Traditional cisterns to store water	
Traditional grape treading	
The Third of May (Goya's painting)	
Bolero Dancing School of Fuente Álamo	
Plaza de la Fuente	
Roman Theatre of Cartagena	
Valle de Ricote	
The Way of Saint James	
La Manga (Mar Menor)	
Pasodoble (Spanish traditional dancing)	
Legend of the lovers of Teruel	
Classical languages (Latin and Greek)	
Pyramids of Egypt	
Great Wall of China	
