# Supplementary material for: Heritage, geographical scale and didactic potentiality: Students and teachers’ perspectives
Source: PLoS One. 2021 May 10;16(5):e0251398. doi: 10.1371/journal.pone.0251398 (PMC8109804; doi:10.1371/journal.pone.0251398)
Supplement: Attachment — Submitted filename: Response to Reviewers - copia.docx [file pone.0251398.s003.docx]

Dear sirs:

With thanks for the recommendations made during the review process about the article “Heritage, geographical scale and didactic potentiality: students and teachers’ perspectives”, we attached to this submission the revised manuscript (with and without track changes).

In addition, the changes made as required are described as follows:

**Reviewer 1**

*Reviewer #1: (…) There are some language/editing issues, such as run-on sentences and grammatical errors, especially noticeable within the abstract and in about the first two pages of the manuscript within the introduction.*

The article has been revised by a professional translator and the changes have been highlighted throughout the paper. In addition, a document signed by the translator on the revision is attached to this resubmission.

*Regarding PLOS ONE's data policy, the data does not seem to be included directly here (i.e. the results of each survey); only statistical summaries of the surveys seem to be included in the manuscript. It would of course be reasonable to not include the base data here considering the data is from a vulnerable population (children), but data availability (or lack thereof) should be more explicitly addressed by the authors considering the publisher's policy. Moreover, though it is discussed and summarized, an example of the actual survey instrument is not included; this would greatly improve the clarity of the discussion.*

Following the indications of the journal, we indicated the availability of anonymized dataset (p. 13., l. 261) and added it to the resubmission (S2 Dataset).

*In general, the statistical methodology is broad-ranging and impressive. However, if I am not misunderstanding, while the initial sample size of students at 506 seems satisfactory, there is a sample size of only 6 teachers. From these 6 teachers, many generalized assumptions are made in the results and interpretation comparing students' to teachers' perspectives as a whole. I'm not sure that such broad generalizations can be reasonably made about teachers in general from only 6 subjects. If, perhaps, there are only 6 teachers of relevant disciplines in the study area, this should be made clear and the potential biases of this small sample enumerated by the authors. It is also not clear if any of these 6 were removed from the final analysis, as it is noted that any incomplete tests were not included, with TOTAL n= 459 after this exclusion (without clarity about the makeup of students vs teachers in this final sample). This needs clarification beyond a general note that the sample is 'intentional non-probabilistic'.*

As the reviewer notes, the initial number of teachers is dependent on the number of teachers in the school in relation to heritage-related areas. This issue is clarified in the paper, as well as the reduction of the sample (p. 9). In addition, we include as part of the discussion the idea that the results cannot be taken with the same robustness in the case of teachers, as the sample is very small (p. 28, l. 530-531).

**Reviewer 2**

*Reviewer #2: The study in the article was presented clearly with terms, research design and execution, and results defined and thoroughly discussed. There are a few minor grammatical errors in the introduction (lines 35, 36, 103) but they do not detract from the clarity of the article. Overall, it presented interesting, original research regarding heritage and provoked questions for further discussion.*

As we pointed out, the article has been revised by a professional translator and the changes have been highlighted throughout the paper. Moreover, a document signed by the translator on the revision is attached to this resubmission.

In addition to the considerations made by the reviewers on language, data availability and sample, we have taken into account the indications made by the editor in relation to the other issues mentioned above, by reviewing:

- The style requirements, including file naming.
- Our reference list.
- The statiscal reporting. All p-values have been changes as “p< .001” where appropriate, as well as other detected changes.

Following the indications of the journal, we indicated the availability of anonymized dataset (p. 13., l. 261) and added it to the resubmission (S2 Dataset).

The Data Availability Statement should be included in the PDF file of the manuscript, as follows:

Anonymized data set is available in this research (S2 Dataset).

We remain at your disposal for any further clarification.

Thank you for your consideration of this manuscript.

Sincerely,

Ana Isabel Ponce
